# H9N2 Influenza Virus Infections in Human Cells Require a Balance between Neuraminidase Sialidase Activity and Hemagglutinin Receptor Affinity

**DOI:** 10.1128/JVI.01210-20

**Published:** 2020-08-31

**Authors:** Yasuha Arai, Emad Mohamed Elgendy, Tomo Daidoji, Madiha Salah Ibrahim, Takao Ono, Nongluk Sriwilaijaroen, Yasuo Suzuki, Takaaki Nakaya, Kazuhiko Matsumoto, Yohei Watanabe

**Affiliations:** a Department of Infectious Diseases, Graduate School of Medical Sciences, Kyoto Prefectural University of Medicine, Kyoto, Japan; b Department of Microbiology and Immunology, Faculty of Veterinary Medicine, Damanhour University, Damanhour, Egypt; c The Institute of Scientific and Industrial Research, Osaka University, Osaka, Japan; d Department of Preclinical Sciences, Faculty of Medicine, Thammasat University, Pathumthani, Thailand; e School of Pharmaceutical Sciences, University of Shizuoka, Shizuoka, Japan; f College of Life and Health Sciences, Chubu University, Aichi, Japan; St. Jude Children's Research Hospital

**Keywords:** H9N2 influenza virus, functional balance, hemagglutinin, host range, infection, neuraminidase stalk

## Abstract

Some avian influenza (AI) viruses have a deletion of up to 20 to 30 amino acids in their neuraminidase (NA) stalk. This has been associated with changes in virus replication and host range. Currently prevalent H9N2 AI viruses have only a 2- or 3-amino-acid deletion, and such deletions were detected in G1 and Y280 lineage viruses, respectively. The effect of an NA deletion on the H9N2 phenotype has not been fully elucidated. In this study, we isolated G1 mutants that carried an 8-amino-acid deletion in their NA stalk. To systematically analyze the effect of NA stalk length and concomitant (de)glycosylation on G1 replication and host range, we generated G1 viruses that had various NA stalk lengths and that were either glycosylated or not glycosylated. The stalk length was correlated with NA sialidase activity, using low-molecular-weight substrates, and with virus elution efficacy from erythrocytes. G1 virus replication in avian cells and eggs was positively correlated with the NA stalk length but was negatively correlated in human cells and mice. NA stalk length modulated G1 virus entry into host cells, with shorter stalks enabling more efficient G1 entry into human cells. However, with a hemagglutinin (HA) with a higher α2,6-linked sialylglycan affinity, the effect of NA stalk length on G1 virus infection was reversed, with shorter NA stalks reducing virus entry into human cells. These results indicate that a balance between HA binding affinity and NA sialidase activity, modulated by NA stalk length, is required for optimal G1 virus entry into human airway cells.

**IMPORTANCE** H9N2 avian influenza (AI) virus, one of the most prevalent AI viruses, has caused repeated poultry and human infections, posing a huge public health risk. The H9N2 virus has diversified into multiple lineages, with the G1 lineage being the most prevalent worldwide. In this study, we isolated G1 variants carrying an 8-amino-acid deletion in their NA stalk, which is, to our knowledge, the longest deletion found in H9N2 viruses in the field. The NA stalk length was found to modulate G1 virus entry into host cells, with the effects being species specific and dependent on the corresponding HA binding affinity. Our results suggest that, in nature, H9N2 G1 viruses balance their HA and NA functions by the NA stalk length, leading to the possible association of host range and virulence in poultry and mammals during the evolution of G1 lineage viruses.

## Abstract (precis)

H9N2 avian influenza (AI) virus, one of the most prevalent AI viruses, has caused repeated poultry and human infections, posing a huge public health risk. The H9N2 virus has diversified into multiple lineages, with the G1 lineage being the most prevalent worldwide. In this study, we isolated G1 variants carrying an 8-amino-acid deletion in their NA stalk, which is, to our knowledge, the longest deletion found in H9N2 viruses in the field. The NA stalk length was found to modulate G1 virus entry into host cells, with the effects being species specific and dependent on the corresponding HA binding affinity. Our results suggest that, in nature, H9N2 G1 viruses balance their HA and NA functions by the NA stalk length, leading to the possible association of host range and virulence in poultry and mammals during the evolution of G1 lineage viruses.

## INTRODUCTION

The currently circulating H9N2 subtype of avian influenza (AI) viruses was first detected in South China in 1994 (1). H9N2 viruses then spread throughout Eurasia and Africa and are now among the most prevalent AI viruses. H9N2 infections cause a decline in egg production with substantial lethality in poultry (2) and also cause a mild respiratory disease in humans (2, 3). Coinfections of H9N2 viruses and other subtype AI viruses frequently generate novel enzootic virus reassortants, e.g., H5N1 A/goose/Guangdong/1/96 (Gs/GD) lineage, H7N9, and H5N8 viruses (1, 4–6). Therefore, it is of great importance to closely monitor possible H9N2 virus genetic and phenotypic changes to reduce the risk of a future pandemic.

The H9N2 viruses that are most prevalent in Eurasia and North Africa are in the G1 and Y280 lineages. G1 lineage viruses have circulated in South China and spread west to Central Asia, the Middle East, and North Africa, where the lineage diversified into four sublineages (sublineages A to D), with sublineages A and B (designated G1-A and G1-B, respectively) being the most prevalent in these areas (7). Y280 lineage viruses have been prevalent throughout China.

In the Middle East, G1-A/B reassortant viruses emerged in Israel and Jordan from 2006 to 2009, with the viral hemagglutinin (HA), neuraminidase (NA), M, and NS genes originating from G1-B and the other viral genes originating from G1-A (7, 8). The G1-A/B virus was first identified in Egypt in 2010 and became endemic in a wide range of poultry (9–11), with four human H9N2 infection cases being reported (2). The G1-A/B virus carried the human adaptation PB2-E627V and -K526R mutations (8), implying that Egypt was a hot spot of H9N2 virus evolution in the Middle East.

Influenza A viruses have two major glycoproteins, hemagglutinin (HA) and neuraminidase (NA), on the virion surface. HA binds to sialylglycan on the surface of a target cell, and NA cleaves the terminal sialic acids (Sia) of sialylglycans to mediate the release of nascent progeny virions from infected cells (12, 13). It has recently been reported that NA also enables virus movement on the cell surface toward endocytic sites, resulting in virus internalization for infection (14, 15). Since both proteins target the same receptor, the functional activities of HA and NA need to be balanced for both virus entry into and virus release from host cells (16).

Human influenza virus HAs preferentially bind the α2,6-linked sialylglycans (α2,6 Sia) that are abundant in human upper airway epithelia, whereas AI virus HAs preferentially bind the α2,3-linked sialylglycans (α2,3 Sia) in bird intestinal epithelia (17). Several AI viruses, including H9N2 G1 lineage viruses, have a relatively high α2,6 Sia binding affinity (18–23), which may facilitate the bird-to-human transmission of AI viruses.

NA is a tetrameric, type II transmembrane protein. The enzyme active site is located in a globular head that is connected to an endodomain via a narrow stalk. The NA stalk length and amino acid sequence vary considerably, even in viruses of the same subtype (24), although there are some common structural features in the stalk, including at least one cysteine residue and a potential glycosylation site. NA stalk deletions have been occasionally detected in AI viruses (e.g., H5N1, H6N1, H2N2, H9N2, H7N1, and H7N3 viruses) and have been associated with strains during early host adaptation from aquatic birds to terrestrial poultry (25–31). The NA stalk length has a significant effect on virus replication, pathogenesis, and even host range for some of these AI viruses.

Experimental passage of a duck H9N2 strain in quail and chickens selected an adapted strain with a 21-amino-acid (aa) deletion in the NA stalk that improved viral replication in two bird species and mice (32). Also, previous studies showed that early isolates of G1 and Y280 lineage viruses in China contained 2- or 3-amino-acid deletions, respectively (33): the 3-amino-acid deletion enhanced the virulence of Y280 lineage virus in chickens and mice (30). However, thus far, the effect of NA stalk length in the G1 lineage viruses now prevalent in Eurasia and Africa on biological changes has not been elucidated.

In this study, we first isolated three H9N2 G1 strains carrying an 8-amino-acid deletion in the NA stalk. This was, to our knowledge, the longest deletion detected naturally in an H9N2 virus. We then investigated the effect of different NA stalk deletions, including the 8-amino-acid deletion, on the G1 virus phenotype. Our results provide data for a better understanding of the effect of NA stalk length on the host range of AI viruses, including G1 lineage viruses.

## RESULTS

### Isolation of H9N2 G1 variants in Egypt with a deletion in the NA stalk.

**FIG 1:**
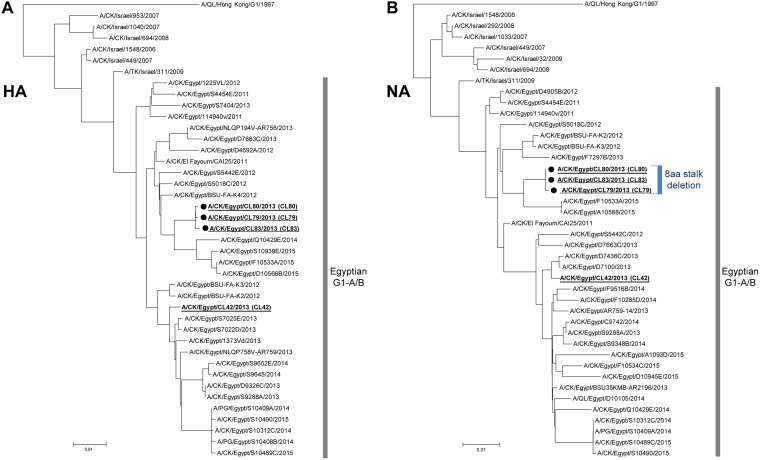
Phylogenetic trees of the HA and NA genes of H9N2 G1 viruses isolated in the Middle East. Phylogenetic trees were reconstructed from the nucleotide sequences of the HA (A) and NA (B) genes of the Middle East reference strains and of the prototype G1 virus using the neighbor-joining method with 1,000 bootstrap replicates. The HA and NA trees were rooted to A/quail/Hong Kong/G1/1997 (H9N2). The three G1 variants isolated in this study (i.e., CL79, CL80, and CL83) with NA stalk deletions are marked with black circles. The CL80 HA and NA genes are representative of those of G1-A/B strains with an 8-amino-acid NA stalk deletion and were used to generate recombinant viruses for this study. The CL42 internal genes are representative of the internal genes of G1-A/B viruses in Egypt and were used to generate recombinant viruses for this study. The CL42, CL79, CL80, and CL83 strains are underlined and in bold font in these trees. CK, DK, QL, and TK in the virus strain names denote chicken, duck, quail, and turkey hosts, respectively.

We collected lung tissue samples from AI virus-infected chickens in the Delta region of Egypt in 2013 and carried out a genetic analysis of the H9N2 viruses in this area. Direct subtyping of AI viruses in lung homogenates showed that three H9N2 strains had a deletion in their NA stalk. These strains (i.e., A/chicken/CL79/2013 [designated CL79], A/chicken/CL80/2013 [designated CL80], and A/chicken/CL83/2013 [designated CL83]) were isolated from different poultry farms by a single passage in chicken embryonated eggs and had the same 8-amino-acid deletion in their NA stalk (58-IERNITEI-65), that included a potential glycosylation site at residues 61 to 63 (underlined). The deleted amino acids were determined from a comparison with the consensus sequence of contemporary H9N2 viruses isolated in Egypt. Phylogenetic analysis of H9N2 G1 viruses isolated in the Middle East showed that the three H9N2 strains with the stalk deletion formed a small G1-A/B sublineage branch in a cluster of other H9N2 viruses prevalent in Egypt (Fig. 1A and B). These results suggested that there may be selective pressure for these G1-A/B viruses to shorten their NA stalk length during virus dissemination in the field.

### Generation of G1-A/B viruses with an NA with various stalk lengths and glycosylation.

**FIG 2:**
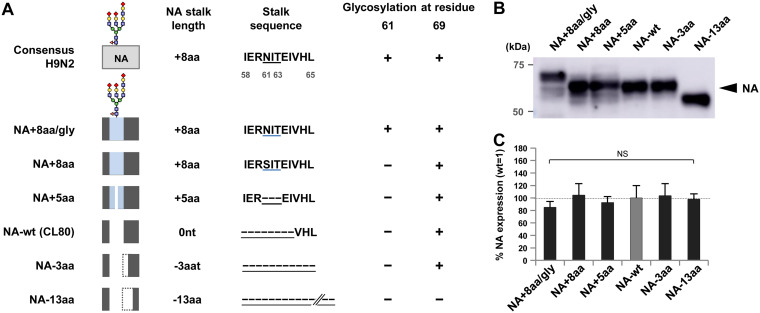
Generation of G1 viruses with various NA stalk lengths. (A) Schematic of the modified CL80 NA with various stalk lengths and glycosylation in this study. The 8-amino-acid deletion (58-IERNITEI-63) in the NA stalk compared to the consensus sequences of the G1 lineage strains in Egypt, including CL42 (consensus H9N2), was found in three G1-A/B strains. NA stalk length differences are shown relative to that of NA-wt. The blue area in the NA boxes indicates an amino acid insertion. A blue underline in the stalk sequences marks residues 61 to 63, which are a potential NA glycosylation site in the consensus H9N2 and NA+8aa/gly strains. A dotted line in an NA box and a black dashed line with an underline in a stalk sequence mark an amino acid deletion. (B) Western blot analysis of the NA mutants with a mobility shift indicating glycosylation in the NA+8aa/gly mutant. A representative Western blot is shown. (C) Quantification of NA expression in 293T cells. The cells were transfected with plasmids expressing the indicated NAs, and cell lysates were analyzed by Western blotting using anti-NA antibody. Each data bar shows the mean ± SD from four independent experiments. nt, nucleotide; NS, no statistically significant difference.

To assess the effect of the NA stalk length deletion on the G1-A/B phenotype, we selected the CL80 strain, which had HA and NA gene sequences that were the most representative of those of the three H9N2 strains isolated in this study with a deletion in their NA stalk. Using a reverse-genetics system, we rescued a recombinant G1-A/B virus (designated the NA wild type [NA-wt]) with HA and NA genes from the CL80 strain and other genes from A/chicken/CL42/2013 (CL42), which had internal gene sequences representative of the G1-A/B lineage strains in Egypt (8, 18). The CL80 NA plasmid was also modified by inserting the 8 amino acids (58-IERNITEI-65) missing in NA-wt (designated NA+8aa/gly) (Fig. 2A). Glycosylation (gly) at residue 61 in NA-wt was confirmed by the mobility shift observed by Western blotting (Fig. 2B). To investigate the effect of glycosylation, a CL80 strain carrying the 8-amino-acid insertion, but with an N61S substitution in the glycosylation site (58-IERSITEI-65, designated NA+8aa), was generated. The N61S substitution was selected based on the sequence of A/quail/Egypt/14864V/2014, which contains no glycosylation site. To differentiate between the effect of glycosylation from the effect of the 3-amino-acid insertion with the glycosylation motif (residues 61 to 63), a CL80 strain with a 5-amino-acid insertion without the glycosylation motif (IER---EI, designated NA+5aa) was generated. A previous study reported that an old H9N2 laboratory strain had lost 21 amino acids in its NA stalk (residues 58 to 83) during experimental passages (32). To systematically analyze the effect of stalk length on G1-A/B replication, two strains containing NA stalk deletions were generated from CL80. One strain had a 3-amino-acid deletion (residues 66 to 68, designated NA−3aa), and the other strain had a 13-amino-acid deletion (residues 66 to 78, designated NA−13aa). The deletion in NA−13aa corresponded to a 21-amino-acid deletion (residues 58 to 83) reported in an experimental passage study (32). All these NAs had equivalent expression levels (Fig. 2C). Also, the result that all these recombinant viruses were successfully rescued indicated that up to a 21-amino-acid deletion in the NA stalk was not deleterious to G1-A/B replication.

### Enzymatic activity of NA stalk length mutants.

**FIG 3:**
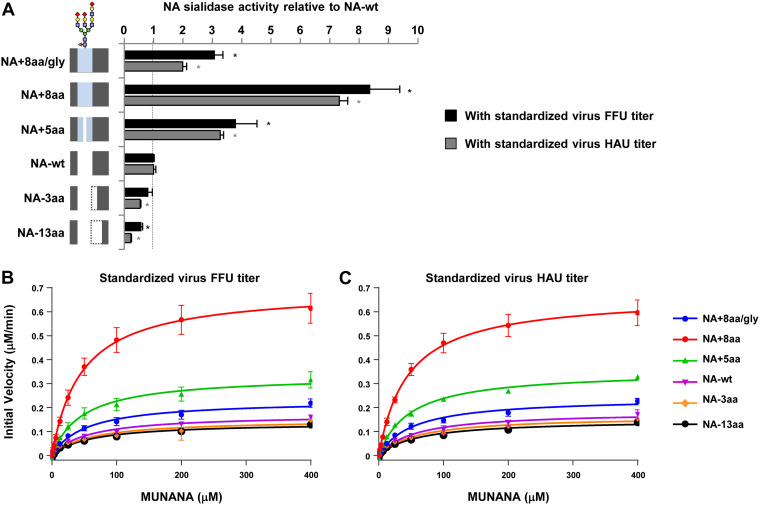
Enzymatic activity of the NA stalk length mutants. (A) The sialidase activity of each NA stalk length mutant was measured using the soluble NA-XTD substrate. Virus samples were standardized to equivalent FFU and HAU titers. NA activities were calculated relative to that of NA-wt. Each data bar shows the mean ± SD from three independent experiments. *, *P* < 0.01. The schematic of the NA stalk in each strain, as shown in Fig. 2A, is shown at the left. (B and C) Enzyme kinetics of the NA stalk length mutants using the soluble MUNANA substrate. (B) Initial reaction velocity with standardized virus FFU titers. (C) Initial reaction velocity with standardized virus HAU titers. The kinetic data for each reaction were fit to the Michaelis-Menten equation by nonlinear fitting to determine the Michaelis-Menten constant (*K_m_*) and the maximum velocity of substrate cleavage (*V*_max_). Each data point is the mean ± SD from three independent experiments with duplicate samples.

The NA activity of NA-wt and the stalk length mutants was determined by measuring the cleavage of the small soluble NA-XTD substrate. Virus samples were standardized to an equivalent titer based on the numbers of focus-forming units (FFU) and on the numbers of hemagglutination units (HAU) and used for the cleavage assays. With both standardized virus titers, NA enzymatic activity varied with the stalk length (Fig. 3A). Relative to the NA activity of NA-wt, NA with the longest NA stalk (NA+8aa) had 9-fold higher activity and NA with the shortest stalk (NA−13aa) had 2-fold lower activity. However, glycosylation at residue 61 significantly reduced the NA activity in NA+8aa/gly, thereby obviating the increase in NA activity produced by the 8-amino-acid insertion in NA+8aa (Fig. 3A, NA+8aa/gly versus NA+8aa).

In addition, the kinetic parameters of the enzymatic activity of the NA stalk length mutants were measured using the fluorescent small 2′-(4-methylumbelliferyl)-α-d-*N*-acetylneuraminic acid (MUNANA) substrate. With NA titers standardized by both the numbers of FFU and the numbers of HAU, NA enzymatic activity varied with stalk length (Fig. 3B and C). NAs with longer stalks had greater enzymatic activity, with the maximum velocity of substrate cleavage (*V*_max_) for NA+8aa being about 4-fold higher than that for NA-wt (Table 1), and the *V*_max_ for NA−13aa was the lowest and slightly less than that for NA-wt. Glycosylation at residue 61 significantly reduced both the enzymatic activity and *V*_max_. All the NA stalk length mutants had relatively similar Michaelis-Menten constant (*K_m_*) values, reflecting their similar substrate binding affinities. Therefore, the relative enzyme efficacies of the NA stalk length mutants, based on the ratios of their *V*_max_ and *K_m_* values, were similar to their relative *V*_max_ values.

**TABLE 1:** Kinetic parameters of enzymatic cleavage of the MUNANA substrate by NA stalk length mutants

Virus	Kinetic parameter value with standardized virus:							
	FFU titer				HAU titer			
	*K_m_* (μM)^*a*^	*V*_max_ (μM/min)^*a*^	*V*_max_ ratio^*b*^	*V*_max_/*K_m_* ratio^*c*^ (relative *E*_eff_^*d*^)	*K_m_* (μM)^*a*^	*V*_max_ (μM/min)^*a*^	*V*_max_ ratio	*V*_max_/*K_m_* ratio (relative *E*_eff_)
NA+8aa/gly	53.2 ± 6.30	0.23 ± 0.008	1.35	1.41	52.7 ± 5.37	0.24 ± 0.008	1.32	1.40
NA+8aa	45.8 ± 4.34	0.69 ± 0.020	4.05	4.92	44.9 ± 3.43	0.66 ± 0.015	3.66	4.55
NA+5aa	47.4 ± 5.64	0.33 ± 0.012	1.95	2.29	48.1 ± 2.78	0.35 ± 0.006	1.93	2.24
NA-wt	55.6 ± 7.02	0.17 ± 0.007	1.00	1.00	55.9 ± 7.95	0.18 ± 0.008	1.00	1.00
NA−3aa	57.7 ± 9.05	0.14 ± 0.011	0.87	0.84	57.9 ± 6.86	0.16 ± 0.006	0.89	0.86
NA−13aa	60.1 ± 7.43	0.13 ± 0.005	0.80	0.74	61.0 ± 7.16	0.14 ± 0.005	0.81	0.74

a The results are the mean ± SD from three independent experiments with duplicate samples.

b The ratio of the *V*_max_ for the NA variants to the *V*_max_ for NA-wt.

c The ratio of the *V*_max_/*K_m_* for the NA variants to the *V*_max_/*K_m_* for NA-wt.

d *E*_eff_, enzyme efficacy.

### Elution of NA stalk length mutants from chicken and turkey erythrocytes.

**FIG 4:**
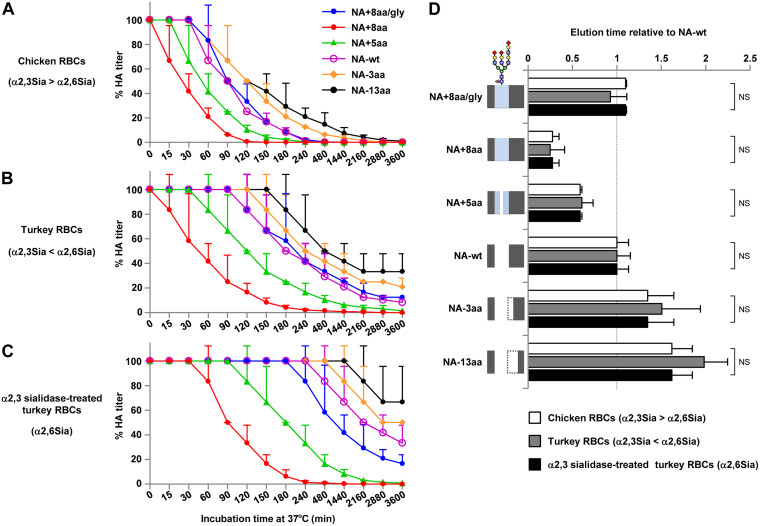
Elution of NA stalk length mutants bound to chicken and turkey erythrocytes (RBCs). (A to C) Twofold dilutions of 128 HAU of each NA mutant were incubated at 4°C for 1 h with an equal volume of chicken erythrocytes (A), turkey erythrocytes (B), and turkey erythrocytes that had been treated with α2,3 neuraminidase (C). The samples were then transferred to 33°C, and their HA titers were assayed as a function of time. The data are expressed relative to the percentage of the initial HA titer at time zero at 4°C. Each data point is the mean ± SD from three independent experiments. The Sia expressed on each type of erythrocyte is shown in parentheses at the left axis. (D) The time when half of each virus eluted from each type of erythrocyte was calculated by fitting the HA elution data with the Boltzmann equation. The data are expressed relative to the results for NA-wt. Each data point is the mean ± SD from three independent experiments. NS, no statistically significant difference. A schematic of the NA stalk in each strain, as shown in Fig. 2A, is shown at the left.

To examine the ability of NA-wt and the NA stalk length mutants to cleave Sia from substrates more complex than MUNANA, we carried out elution assays with viruses bound to chicken and turkey erythrocytes. Chicken erythrocytes express significantly more α2,3 Sia than α2,6 Sia, whereas turkey erythrocytes express significantly more α2,6 Sia than α2,3 Sia (34). Therefore, erythrocytes from both avian species were used to assay NA substrate specificity. The elution efficacy from these two erythrocyte species was in agreement with our results on the effect of NA stalk length on NA enzymatic cleavage of the MUNANA substrate (Fig. 4A and B); i.e., NA+8aa, with the longest stalk, eluted the most quickly, whereas NA−13aa, with the shortest stalk, eluted the most slowly. Glycosylation of residue 61 largely blocked the increased elution produced by the 8-amino-acid insertion (Fig. 4A and B, NA+8aa/gly versus NA+8aa). All the viruses eluted more slowly from turkey erythrocytes, which mostly expressed α2,6 Sia, than from chicken erythrocytes, which mostly expressed α2,3 Sia. This difference was greater when turkey erythrocytes were treated with α2,3 sialidase, so that only α2,6 Sia was on the cell surface (Fig. 4C). This result was in agreement with the results of previous studies showing that AI virus NAs have a cleavage preference for α2,3 Sia (35). The time to elute half of the bound viruses was calculated by curve fitting the nonlinear elution data and expressing the data relative to those for NA-wt. The relative elution times were almost identical for all three types of erythrocytes (Fig. 4D), implying that NA stalk length did not affect the substrate specificity for Sia cleavage. Collectively, these results suggest that NA stalk length is correlated with enzymatic activity and elution efficacy from erythrocytes but not with substrate specificity.

### Replication of NA stalk length mutants in avian cells and eggs.

**FIG 5:**
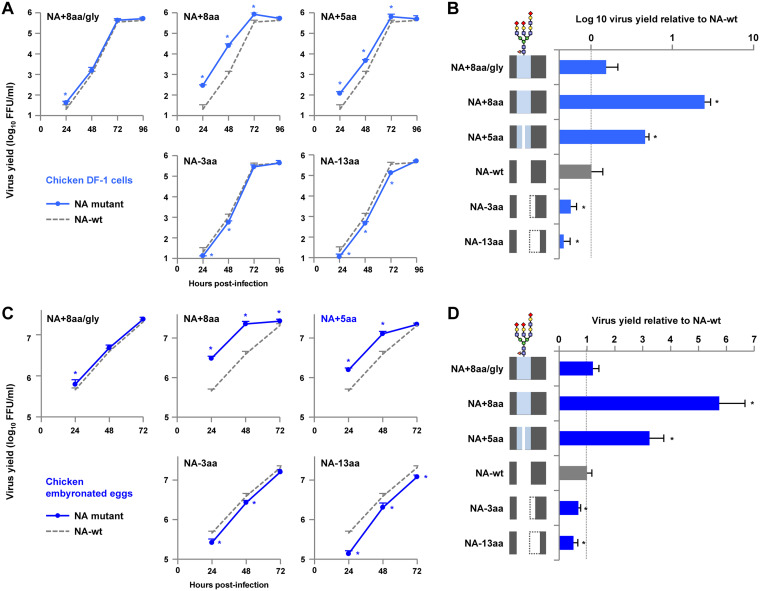
Replication of NA stalk length mutants in an avian cell line and eggs. (A and C) Growth kinetics of NA mutants in avian cells and eggs. (A) Chicken DF-1 cells were infected with the indicated viruses at an MOI of 0.005 and cultured at 37°C for 96 hpi. (C) Nine-day-old embryonated chicken eggs (5 eggs per group) were inoculated with 1 × 10^5^ FFU of the indicated viruses and incubated at 37°C for 72 hpi. The culture supernatants and allantoic fluids were harvested at the indicated times and assayed for the numbers of FFU to determine the progeny virus titers. (B and D) Relative virus yields at 48 hpi. The virus titers produced by the NA mutants in DF-1 cells (B) and eggs (D) are shown relative to the titers produced by NA-wt. Each data point is the mean ± SD from three independent experiments. *, *P* < 0.01. A schematic of the NA stalk in each strain, as shown in Fig. 2A, is shown at the left.

The effect of stalk length on the replication of NA stalk length mutants in avian cells and eggs was investigated. Chicken-derived DF-1 cells and embryonated eggs were infected with NA-wt and the NA stalk mutants, and their replication kinetics at 37°C were measured. Virus replication in DF-1 cells and eggs was closely correlated with NA stalk length: the longer that the stalk was, the better that the replication was (Fig. 5A and C). The virus titers early postinfection were NA+8aa > NA+5aa > NA-wt > NA−3aa > NA−13aa. At 48 h postinfection (hpi), NA+8aa, with the longest NA stalk length, produced 24.4- and 5.7-fold higher progeny virus titers in DF-1 cells and eggs, respectively, than NA-wt (Fig. 5B and D). In contrast, NA−13aa, with the shortest NA stalk length, produced less than half the progeny virus yield of NA-wt. Glycosylation at residue 61 significantly decreased the increase in G1-A/B replication due to the 8-amino-acid insertion (Fig. 5B and D, NA+8aa/gly versus NA+8aa).

### Replication of NA stalk length mutants in a human cell line and primary cells.

**FIG 6:**
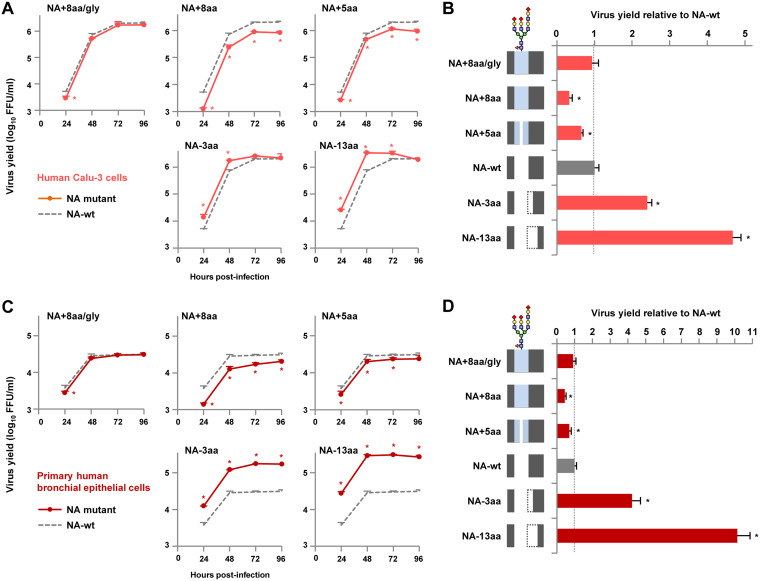
Replication of NA stalk length mutants in a human cell line and primary airway cells. (A and C) Growth kinetics of NA mutants in human Calu-3 cells (A) and primary human bronchial epithelial cells (C). The cells were infected with the indicated viruses at an MOI of 0.03 and cultured at 33°C for 96 hpi. The culture supernatants were harvested at the indicated times and assayed for the numbers of FFU to determine the progeny virus titers. (B and D) Relative progeny virus titers at 48 hpi. The virus titers produced by the NA mutants in Calu-3 cells (B) and primary human airway cells (D) are expressed relative to the titers produced by NA-wt. Each data point is the mean ± SD from three independent experiments. *, *P* < 0.01. A schematic of the NA stalk in each strain, as shown in Fig. 2A, is shown at the left.

To investigate the relationship between NA stalk length and G1-A/B mutant replication in human airway cells, human airway-derived Calu-3 cells and primary bronchial epithelial cells were infected and viral replication kinetics at 33°C were measured for 96 h. In contrast to the growth kinetics in avian cells, the replication of NA stalk length mutants in human cells was negatively correlated with the NA stalk length (Fig. 6A and C). The relative progeny virus titers were NA−13aa > NA−3aa > NA-wt > NA+5aa > NA+8aa. At 48 hpi, NA−13aa grew efficiently, with progeny virus yields in Calu-3 cells and primary cells being 4.8- and 10.1-fold higher than those for NA-wt, respectively (Fig. 6B and D). In contrast, NA+8aa had the lowest progeny virus yields in Calu-3 cells and primary cells, 3.0- and 2.3-fold lower than those for NA-wt, respectively. Glycosylation at residue 61 blocked the increase in G1-A/B replication in human cells due to the 8-amino-acid insertion (Fig. 6B and D, NA+8aa/gly versus NA+8aa).

Taken together, these results indicate that NA stalk deletions lead to an increase in virus replication in human airway cells but a decrease in avian cells infected with the G1-A/B mutants. These results also indicate that the NA stalk length has an appreciable effect on host adaptation of the G1-A/B virus *in vitro*.

### Pathogenicity of NA stalk length mutants in mice *in vivo*.

To assess the effects of changes in NA stalk length on *in vivo* infections, BALB/c mice were inoculated intranasally with 10^3^ to 10^7^ FFU of either NA-wt or one of three representative stalk length mutants, i.e., NA+8aa/gly, NA+8aa, or NA−13aa. The body weight and survival of each infected mouse were monitored daily for 14 days.

**FIG 7:**
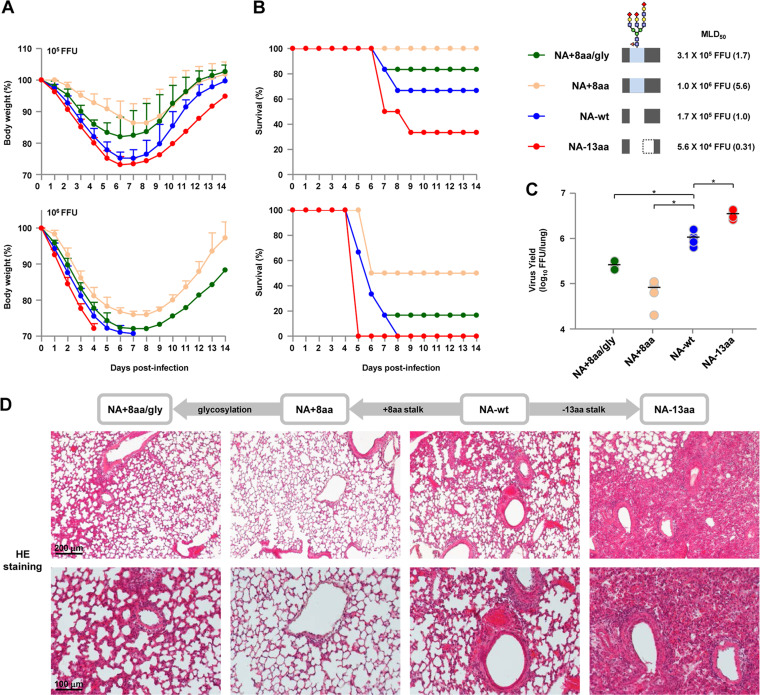
Virulence and *in vivo* replication of NA stalk length mutants in mice. (A and B) BALB/c mice (6 mice per group) were inoculated intranasally with 10^5^ (top) or 10^6^ (bottom) FFU of NA-wt and or an NA mutant virus with the indicated NA stalk length. (A) The body weights of the infected mice were monitored daily for 14 dpi. The mean ± SD of the percentage of the initial body weight for each group of mice is shown. (B) Survival of the infected mice. Survival was calculated for all mice, including mice that were humanely sacrificed if they lost more than 30% of their body weight within a few days. A schematic of the NA stalk of each strain (as shown in Fig. 2A), the MLD_50_ of that strain, and its value relative to that for NA-wt (in parentheses) are shown at the right. (C) Virus titers at 4 dpi in the lungs of mice (5 mice per group) infected with 10^5^ FFU of the indicated viruses. Each symbol marks the titer in an individual mouse. *, *P* < 0.01. (D) (Top row) Representative low-magnification photomicrographs at 4 dpi of hematoxylin- and eosin (HE)-stained sections of lungs from mice infected with the indicated viruses. (Bottom) Magnified views of the photomicrographs in the top row. The different NA stalk lengths in the viruses are shown above panel D.

With an inoculation dose of 10^5^ FFU, the NA+8aa virus, with the longest stalk length, caused a small weight loss in all 6 infected mice, and all of the infected mice survived (Fig. 7A and B). In contrast, the same dose of NA-wt and NA−13aa viruses caused severe weight loss; 4 of the 6 mice infected with NA-wt survived, and 2 of the 6 mice infected with NA−13aa survived. With a dose of 10^6^ FFU, there was significant weight loss in all the infected mice; 3 of the 6 mice infected with NA+8aa survived, but none of the mice infected with NA-wt or NA−13aa survived, with death being faster for NA−13aa-infected mice than for NA-wt-infected mice. NA−13aa was the most pathogenic in mice, with its 50% mouse lethal dose (MLD_50_) being 3.2-fold lower than that of NA-wt, whereas NA+8aa was the least pathogenic, with its MLD_50_ being 5.6-fold higher than that of NA-wt. Glycosylation at residue 61 increased the pathogenicity effect of the 8-amino-acid stalk insertion, resulting in rapid weight loss and the reduced survival of infected mice (NA+8aa versus NA-wt).

To determine the virus titers in infected mice, groups of five mice were inoculated intranasally with 10^5^ FFU of each virus and their lungs were collected at 4 days postinfection (dpi). The virus titers in the infected mice were in agreement with their clinical signs and with the *in vitro* replication ability of the virus in human airway cells. The progeny virus yield in the lungs of mice infected with NA−13aa was considerably higher than that in the lungs of mice infected with NA-wt (Fig. 7C). In contrast, NA+8aa had the lowest progeny virus yield in the lungs of infected mice. Glycosylation at residue 61 produced an intermediate progeny virus yield in the lungs of NA+8aa/gly-infected mice, between that of NA-wt-infected mice and NA+8aa-infected mice.

Pathology studies were carried out on the lungs of infected mice collected at 4 dpi. NA+8aa produced only a limited inflammatory response in the lungs of infected mice (Fig. 7D). In contrast, NA−13aa induced more severe pathological changes in the lungs than NA+8aa did, with bronchiolar necrosis and alveolitis characterized by hemorrhage and infiltration of inflammatory cells. NA-wt and NA+8aa/gly caused moderate pneumonia in infected mice, with slightly less severe bronchiolar hemorrhage and inflammatory cell infiltrates being seen in NA+8aa/gly-infected mice than in NA-wt-infected mice. Together, these results indicate that the NA stalk deletion enables increased viral replication in human airway cells *in vitro* and contributes to increased replication in infected mice *in vivo*, which suggest that the NA stalk length has appreciable effects on the mammalian adaptation of G1-A/B NA mutants.

### Effect of the balance between HA and NA functions on virus infection of human cells.

The balance between HA and NA functions, which has been reported to be critical for influenza virus replication and host range (14, 15), was investigated using the NA mutants in this study. We first measured HA binding affinity to the α2,3 Sia and α2,6 Sia analogues Neu5Acα2,3Galβ1-4GlcNAc (α2,3 SLN) and Neu5Acα2,6Galβ1-4GlcNAc (α2,6 SLN), respectively, and to α2,3 SLN sulfated on its antepenultimate sugar [(Neu5Acα2,3Galβ1-4(6-HSO_3_)GlcNAc (Su-α2,3 SLN)], which has been reported to be recognized by H9N2 G1 viruses (21, 36). In this study, we used three H9N2 G1 strains that were isolated in the Middle East (i.e., CL80, CL42 and A/chicken/Emirates/R66/02 [designated Em/R66]), each of which had an HA with a distinct Sia binding pattern (described below).

**FIG 8:**
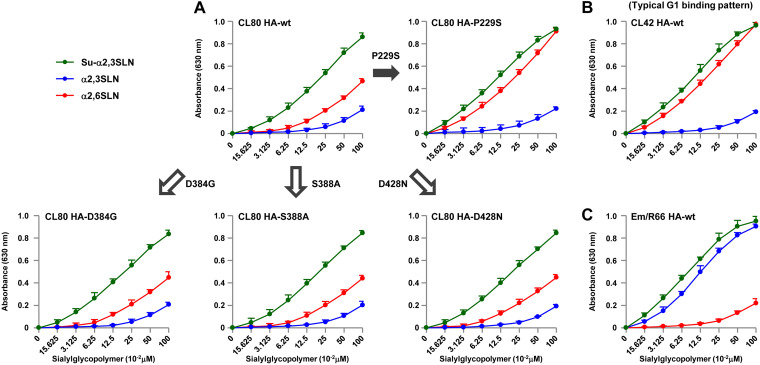
Receptor binding specificity of the CL80 HA, CL42 HA, Em/R66 HA, and mutant HAs. (A to C) Binding assays of the indicated viruses to three receptor analogues: α2,6 SLN, α2,3 SLN, and sulfated α2,3 SLN (Su-α2,3 SLN). CL42 HA is representative of a G1 lineage HA with high α2,6 Sia binding. Each data point is the mean ± SD from three independent experiments.

CL80 HA, a representative G1-A/B mutant with a shortened NA, had strong binding to Su-α2,3 SLN, moderate binding to α2,6 SLN, and low binding to α2,3 SLN (Fig. 8A). However, relative to CL80 HA, CL42 HA had significantly increased binding to α2,6 SLN and slightly increased binding to Su-α2,3 SLN (Fig. 8B). The CL42 HA binding pattern was similar to that of contemporary G1 viruses (20). The ectodomain sequence of the CL80 HA (without the signal peptide) differed from that of the CL42 HA, with substitutions at eight HA amino acid residues. Among these differences, only the P229S mutation was located in the receptor binding domain, implying its putative association with the Sia binding difference between the two viruses. To confirm the effect of the P229S mutation in enabling increased α2,6 Sia binding of CL42 HA, viruses carrying CL80 HAs with a single P229S mutation were rescued and the effects of the mutation on Sia binding were measured. As controls, we arbitrarily selected three of the remaining seven mutations that were located in the HA stalk domain (i.e., D384G, S388A, and D428N) and rescued viruses with these three mutations. The D384G, S388A, and D428N reverse mutations had little effect on Sia binding by CL80 HA (Fig. 8A and B). However, the P229S reverse mutation considerably increased α2,6 SLN binding and slightly increased Su-α2,3 SLN binding, resulting in a binding pattern resembling that of CL42 HA. This indicated that the S229P mutation appreciably reduced CL80 HA binding to α2,6 SLN. However, Em/R66 HA showed an atypical Sia binding pattern, with strong binding to both α2,3 SLN and Su-α2,3 SLN and low binding to α2,6 SLN (Fig. 8C), as previously reported (21). These results suggest that G1-A/B variants with a shortened NA have a fairly restricted α2,6 Sia binding affinity but strong binding to α2,3 Sia (mostly sulfated α2,3 Sia).

Our data indicate that the G1-A/B variants had HAs with a poor α2,6 Sia affinity and, therefore, needed NAs with reduced sialidase activity for more efficient growth in human cells (Fig. 3, 6, and 8). These results suggest that reduced NA activity, due to the stalk length deletion, compensated for the limited HA binding affinity to improve virus growth in human cells. Recent studies reported that the HA and NA functions needed to be modulated to produce a balance in their activities that was required for virus entry into target cells (14, 15). Therefore, we investigated whether the effect of NA stalk length on the infection of human airway cells by G1-A/B variants was related to the relatively low α2,6 Sia binding affinity of the HAs of these viruses.

**FIG 9:**
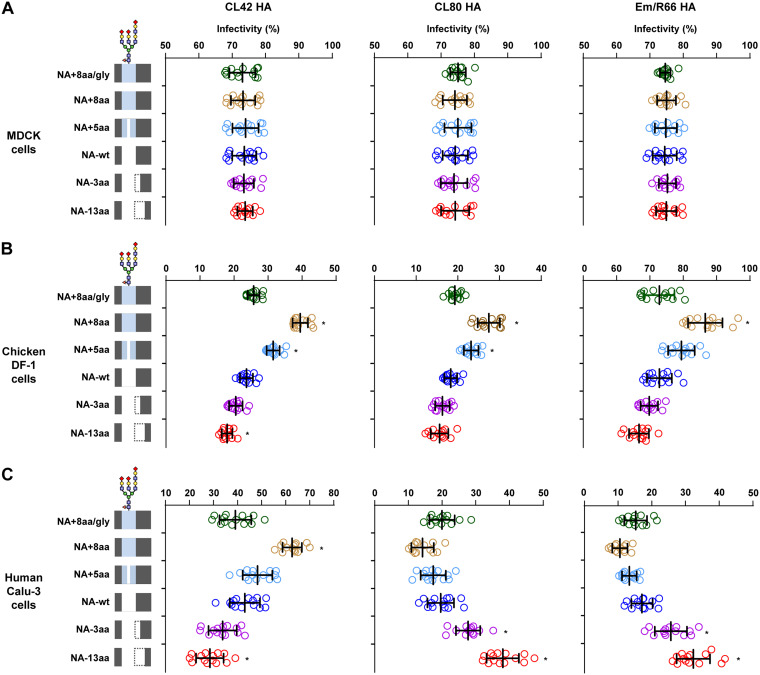
Infectivity of G1 viruses with reassorted HA/NA in cultured cells. Semiconfluent monolayers of cells grown in 96-well plates were infected with G1 viruses carrying an NA mutant with a modified stalk and an HA from CL80 (left), CL42 (middle), or Em/R66 (right). The MOIs of these infections were equalized by FFU assays on MDCK cells. (A) MDCK cells; (B) DF-1 cells; (C) Calu-3 cells. After 1 h of adsorption and washing, the cells were overlaid with 1% methylcellulose in medium without trypsin, to prevent the release of progeny virus, and analyzed by immunofluorescence assays. Virus infections were assayed by counting virus antigen-positive cells and cell nuclei in the same field with an IN Cell analyzer automated microscope. Data are expressed as the mean ± SD from 14 independent experiments, with the results of individual experiments shown as open symbols. *, *P* < 0.01. A schematic of the NA stalk in each strain, as shown in Fig. 2A, is shown at the left.

To investigate how the balance of HA and NA functions affected virus entry into host cells, we generated viruses carrying either NA-wt or 1 of our 5 NA stalk length mutants and an HA with a distinct Sia affinity from CL80, CL42, or Em/R66. The multiplicities of infection (MOIs) for the virus infections were equalized based on FFU assays on MDCK cells. The infectivity of these viruses was measured with an IN Cell analyzer automated microscope, which enabled us to accurately quantify high numbers of foci in image fields (37, 38). In this study, after virus adsorption, the cells were washed and then overlaid with 1% methylcellulose in medium without trypsin to prevent the release and spread of progeny virus, thereby enabling us to observe only foci that were a result of the initial input virus infection. Most H9N2 G1 lineage viruses, including CL80, CL42, and Em/R66, had HAs with mono- or dibasic cleavage sites, and their growth depended on trypsin (20, 39). The viruses showed identical infectivity on MDCK cells (Fig. 9A), which confirmed the equalization of the MOIs used for this study. DF-1 cells were then incubated with equal virus MOIs, and infectivity was quantified. The results showed that the infectivity of viruses with an HA from either CL42, CL80, or Em/R66 was correlated with the NA stalk length in these avian cells (Fig. 9B). This was in agreement with our previous result that these viruses had substantially strong binding to α2,3 Sia-terminated glycans (α2,3 SLN and/or Su-α2,3 SLN) (Fig. 8). These results indicate that efficient host cell infection by viruses carrying HA with a high Sia affinity needs to be balanced by an NA with high sialidase activity. Calu-3 cells were then incubated with these viruses, and infectivity was quantified. As was found for DF-1 cells, the infection of human Calu-3 cells by viruses carrying CL42 HA with a high α2,6 Sia binding affinity was correlated with the NA stalk length in those viruses: the highest infectivity was with viruses carrying an NA with the longest stalk (Fig. 9C). In contrast, the infectivity of Calu-3 cells with CL80 or Em/R66, which have an HA with a low binding affinity, was negatively correlated with the NA stalk length: virus infectivity was the highest with an NA with a short stalk length and the lowest with an NA with a long stalk length. These results indicate that the effect of NA stalk length on infectivity depends on the binding affinity and/or Sia specificity of the corresponding HA. The results also suggest that a low HA binding affinity can be compensated for by reduced NA activity due to an NA stalk deletion, thereby increasing the infectivity of G1-A/B mutants in human airway cells. As was observed in our previous results (Fig. 3 and 7), glycosylation at NA residue 61 largely canceled the effect of NA stalk length on viral infectivity. Since the effect of NA stalk length on infectivity was similar in viruses carrying CL80 HA or Em/R66 HA, infectivity was the result of viral HA affinity for an α2,6 Sia- or α2,3 Sia-terminated glycan but not for a sulfated Sia. In summary, these results indicate that the balance between HA and NA activities determines virus entry and the host range of G1 lineage AI viruses.

### Effect of NA stalk length on progeny virus release.

**FIG 10:**
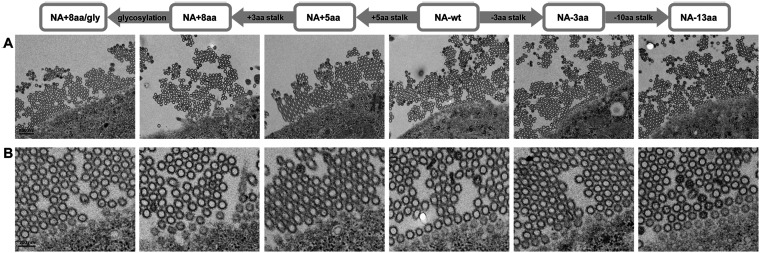
Progeny virus release of NA stalk length mutants. (A) Representative transmission electron micrographs of thin sections of MDCK cells infected by the indicated viruses (low magnification). (B) High magnification of the micrographs in panel A, showing progeny virions budding from the cell surface and virus aggregates.

Finally, we investigated the effect of NA stalk length on progeny virus release by electron microscopy of infected MDCK cells. The micrographs showed that all the NA stalk length mutants had relatively comparable levels of progeny virus aggregates with a similar morphology on the surfaces of the infected cells (Fig. 10). These results suggest that the NA stalk length mutants in this study do not appear to affect the release of progeny virions.

## DISCUSSION

The balance between the HA and NA functions is a determinant of influenza virus growth, virulence, and host range (40–42). NA stalk deletions and deglycosylation in the NA stalk can modify the HA/NA balance, thereby changing the virus phenotype. Several studies have reported some effects of HA deletions on the H9N2 properties of an old laboratory A/duck/Hong Kong/702/79 strain and of an ancestral Y280 A/chicken/Shandong/16/05 strain (30, 32). However, there have been no studies, thus far, analyzing the effect of NA deletions on biological changes in the G1 lineage strains that are now the most prevalent AI virus strains in Eurasia and Africa. To our knowledge, this is the first report showing a relationship between the NA stalk length, viral growth, and host adaptation of G1 lineage viruses. A similar type of HA/NA balance may be significant for infections of other AI virus lineages.

The NA stalk length affects its enzymatic activity by modifying the structural flexibility of the NA enzymatic pocket (43) and/or its accessibility to Sia (44, 45). NA stalk deletions have been reported in several subtype AI viruses and have been associated with viral adaptation in mammalian species (25–27, 29, 32, 46). The H5N1 Gs/GD lineage has a 19- to 20-amino-acid deletion in its NA stalk, which reduces NA enzymatic activity and increases viral virulence in mice (28, 31). Experimental deletion of 21 amino acids in the NA stalk contributed to the higher mouse pathogenicity of an old H9N2 strain (32). In this study, 3- to 21-amino-acid deletions in the NA stalk increased G1-A/B growth in human cells and mice in a deletion-dependent manner. The NA stalk deletions also reduced both NA enzymatic activity with small substrates and viral elution efficacy from erythrocytes, suggesting that adaptation of virus growth in mammals involves a reduction of NA enzymatic activity due to stalk deletions. This was in agreement with the findings of previous studies of other AI viruses (28, 31, 32).

The role of NA stalk glycosylation on virus properties still needs to be fully elucidated. NA glycosylation has been suggested to contribute to virus replication via altered antigenicity and/or enzymatic activity (47). This study showed that glycosylation of NA stalk residue 61 reduced NA enzymatic activity and blocked the increase in NA activity in an NA with an 8-amino-acid insertion in its NA stalk (residues 58 to 65). NA+8aa/gly had less enzymatic activity than NA+5aa, although NA+8aa/gly had a 3-amino-acid longer stalk (longer by the NIT residues). This implied that glycosylation at residue 61 blocked the increase in NA activity that should have been due to the 3-amino-acid insertion. Thus, NA stalk glycosylation had a strong impact on NA enzymatic activity. This was in agreement with previous studies showing that a 3-amino-acid deletion (i.e., the glycosylation motif at residues 61 to 63) affected the phenotype of Y280 lineage A/chicken/Shandong/16/05 (30, 33). Also, the increased virulence of A/Anhui/01/2013 (H7N9) was attributed to a 9-amino-acid NA stalk deletion (from residues 57 to 65), which included the glycosylation motif (48), but not the adjacent 5 amino acids (residues 69 to 73), which did not include a glycosylation motif (49). There have been reports of the conflicting effects of short stalk deletions on virus properties. However, our data suggest that a short NA stalk deletion, even a 3-amino-acid deletion, has a considerable effect on NA enzymatic activity and on the virus phenotype, if the deletion includes the glycosylation motif. Collectively, these results suggest that both stalk length and (de)glycosylation contribute to NA enzymatic activity and virus properties.

Recent studies reported that NA plays a role at the cell surface in virus entry. Virions attach to the cell surface by HA binding to Sia, but most Sia does not mediate virion endocytosis for virus entry. Viruses migrate on the cell surface to target specific domains with preexisting components of the endocytic pathway (14). NA enzymatic activity is needed to drive virion rolling by creating a Sia gradient that forces a virus to roll away from inappropriate Sia receptors (15). HA and NA act cooperatively to move virions on the cell surface. If the HA and NA activities are not balanced and the NA activity is suboptimal, the virion remains stacked on the cell surface, preventing virus movement and entry. Conversely, when NA activity is too strong compared to the HA binding affinity, virion binding is disrupted by NA activity, resulting in detachment (14). Thus, a balance in HA and NA activities drives optimal virion movement on the cell surface, thereby accelerating virus internalization.

In this study, the G1-A/B mutants carried a shortened NA with low sialidase activity that contributed to their efficient growth in human airway cells (Fig. 6), although it has been suggested that lower NA activity is a disadvantage for progeny virus release. The different NA stalk lengths in this study did not have a noticeable effect on progeny virus yield (Fig. 10). In addition, viruses with a shorter NA stalk retained some elution activity from chicken erythrocytes with α2,3 Sia abundance (Fig. 4A), which resembled the expression pattern of mucin. This implies that shortened NAs are still capable of penetrating mucin layers.

Our study shows that NA stalk length significantly affects virus entry into human airway cells and that the effect of NA stalk length on viral infection depends on the corresponding HA binding affinity/specificity for target cells (Fig. 9). Collectively, our results suggest that reduced NA activity due to an NA stalk deletion compensated for the reduced HA binding and acted to optimize G1-A/B mutant virus infection of human airway cells, perhaps by modulating virion rolling/uptake on the cell surface. Future studies are needed to fully investigate the effect of NA stalk length on the efficacy of release of progeny G1 viruses with a modified NA stalk length.

In this study, G1-A/B mutants with an 8-amino-acid deletion in their NA were isolated from different, but geographically close, poultry farms in Egypt. This indicates that the G1-A/B mutants with a shortened NA were replication competent and had disseminated in the local poultry population. However, these G1-A/B mutants have not been subsequently detected in the field. Contemporary Egyptian G1-A/B viruses have an NA stalk motif identical to that of NA+8aa/gly, i.e., NA-wt with an 8-amino-acid insertion and glycosylation at residue 61. Viruses carrying NA+8aa/gly had higher NA sialidase activity (Fig. 3) and slightly more efficient growth in avian cells and eggs than viruses carrying NA-wt (Fig. 5). Thus, the G1-A/B mutants appeared to have no replicative advantage to enable them to replace the prevalent G1-A/B strains in the avian population, which would have led to the loss of the mutant strains. NA stalk glycosylation could implicate an antigenic change (47), suggesting selective pressure for G1-A/B mutants to lose glycosylation to escape immune pressure in the local poultry population. This may explain why a mutant virus with deglycosylated NA emerged in the field, despite its lower replication in poultry.

Since currently prevalent H9N2 G1 lineage viruses can balance their HA and NA functions, their ongoing propagation may promote viral genetic and phenotypic diversification in the field, providing many opportunities to alter the HA/NA balance. During influenza virus adaptation to human hosts in past pandemics, reduced HA activity may have been compensated for by decreased NA activity (50). Therefore, balancing HA and NA functions, particularly if NA contains a stalk deletion, may produce a future G1 lineage virus adapted for mammalian infection. The results presented here provide a novel insight into the functional interdependence of HA and NA on the infectivity and host range of influenza viruses and highlight the importance of closely monitoring the possible emergence of H9N2 G1 mutants with an expanded host range in areas of endemicity.

## MATERIALS AND METHODS

### Ethics statement.

Nine-day-old embryonated chicken eggs were purchased from Shimizu Laboratory Supplies, Japan. All animal experiments were conducted in compliance with Japanese legislation and guidelines under the jurisdiction of the Ministry of Education, Culture, Sports, Science and Technology in Japan. Animal care, housing, feeding, sampling, observation, and environmental enrichment were approved by the Animal Experiment Committee of the Kyoto Prefectural University of Medicine (approval numbers M29-554 and M30-60).

### Biosecurity and biosafety.

All experiments with live H9N2 viruses were performed at enhanced biosafety level 3+ facilities in the Kyoto Prefectural University of Medicine. All studies with recombinant DNA were conducted under the relevant laws in Japan and approved by the Biological Safety Committee of the Kyoto Prefectural University of Medicine (approval number 30-104) after risk assessments were conducted by the Living Modified Organisms Committee of the Kyoto Prefectural University of Medicine and, when required, by the Ministry of Education, Culture, Sports, Science and Technology of Japan.

### Cells and viruses.

Human bronchial epithelial (Calu-3) cells and chicken fibroblast (DF-1) cells were obtained from several cell banks and maintained as described previously (8, 18). Primary human bronchial epithelial cells were obtained from PromoCell and maintained according to the manufacturer’s recommendations. None of the cell cultures in this study were grown or infected under air-liquid interface conditions as described previously (51–53). Influenza virus A/chicken/Egypt/CL42/2013 (CL42) (H9N2) is a representative strain of the G1-A/B lineage that has been prevalent in Egypt and that has been described previously (18). All recombinant H9N2 viruses were propagated once in 10-day-old embryonated eggs and purified by ultracentrifugation as described previously (8, 52).

### Virus sampling and subtyping.

Lung tissue samples were collected from sick and dead chickens from different poultry farms during AI outbreaks in the Delta region of Egypt in 2013. Viral RNAs were extracted directly from lung homogenates and analyzed by reverse transcription-PCR using the Uni-12 RT primer and primer sets specific for HA or NA genes, as described previously (52, 54). The PCR products were analyzed by agarose gel electrophoresis. Specific gel bands were purified with a QIAquick gel extraction kit (Qiagen) and ligated into the pCR-TOPO vector with a TA cloning kit (Invitrogen) according to each manufacturer’s instructions. For each virus, the nucleotide sequences of the HA and NA genes of at least 5 clones were aligned using the Genetyx (version 11) program (Genetyx Corp.) to determine the dominant sequences. After subtyping based on direct sequence analysis, viruses were isolated by single passage in the allantoic cavity of 10-day-old embryonated chicken eggs.

### Phylogenetic analysis.

The sequences of viral HA and NA gene segments from reference H9N2 strains, isolated from 2006 to 2015, were obtained from the GISAID database (https://www.gisaid.org). Phylogenetic analysis was performed by the neighbor-joining method using Mega (version 6) software with the nucleotide sequences of representative H9N2 strains, the CL42 strain, and the CL79, CL80, and CL83 strains isolated in this study. The confidence levels for the phylogenetic trees were calculated by performing 1,000 bootstrap replicates.

### Generation of recombinant viruses.

Recombinant viruses were generated with a plasmid-based reverse genetics system as described previously (55, 56). Plasmids encoding CL80 NA-wt, the NA stalk mutants, and HAs from CL80, CL42, or Em/R66 were used with plasmids carrying the other viral genes from the CL42 virus. The NA and HA mutations were determined by comparing the nucleotide sequences of the H9N2 strains in this study with the consensus sequences of contemporary H9N2 viruses in Egypt. Mutations were introduced into the plasmids in this study using PCR-based site-directed mutagenesis. All the propagated viruses were completely sequenced to ensure the absence of unwanted mutations.

### Virus titration by focus-forming assays.

Confluent monolayers of MDCK cells in 96-well plates were infected, and after 1 h at 37°C, the virus inoculum was removed and the cells were washed twice with phosphate-buffered saline (PBS), overlaid with 1% methylcellulose in modified Eagle’s medium without trypsin, and incubated at 37°C. At 12 h postinfection, the cells were fixed with 4% paraformaldehyde in PBS, and the numbers of focus-forming units (FFU) were determined by immunofluorescence microscopy, as described previously (23, 51, 56). Foci were counted using an inverted fluorescence microscope system (Eclipse Ti2; Nikon).

### Western blotting.

293T cells were transfected with equal amounts of plasmids encoding CL80 NA-wt and the stalk variants. At 16 h posttransfection, the cells were harvested with sample buffer. The samples were analyzed by SDS-PAGE and transferred onto polyvinylidene difluoride membranes (Millipore). Immunoblot analysis was performed using anti-influenza virus H9N2 NA antibody (Sino Biological) and horseradish peroxidase-conjugated secondary antibody. The bands were visualized with the Amersham ECL Select Western blotting detection reagent and an Amersham Imager 600 (GE Healthcare). The band intensities were quantified by Amersham Imager 600 Analysis software (GE Healthcare).

### NA enzyme activity.

The NA activity of each virus was determined based on cleavage of the small soluble NA-XTD substrate using an NA-XTD influenza virus neuraminidase assay kit (Thermo Fisher Scientific) following the manufacturer’s instructions. Virus samples were standardized to an equivalent dose of 3.4 × 10^7^ FFU or 51.2 HAU. A series of 2-fold-diluted virus samples was mixed with NA-XTD substrate in 50 μl NA-XTD assay buffer. The reaction mixtures were incubated at room temperature for 20 min, and chemiluminescence was measured using an SH-9000 lab microplate reader (Corona Electric). The virus dilution corresponding to the midpoint of the linear part of the sigmoidal curve of NA activity was chosen to determine the relative NA cleavage activity of the NA-XTD substrate. NA activities were expressed relative to the activity of NA-wt, which was set at 100%.

### NA enzymatic kinetics.

NA enzymatic parameters were measured using the fluorescent 2′-(4-methylumbelliferyl)-α-d-*N*-acetylneuraminic acid (MUNANA; Sigma-Aldrich) substrate by a procedure similar to that described in previous reports (57, 58). Briefly, virus samples were standardized to an optimized titer of 3.3 × 10^5^ FFU or 12.8 HAU and mixed with MUNANA (final concentration, 0 to 400 μM) at pH 6.5 in a reaction buffer containing 32.5 mM 2-(*N*-morpholino)ethanesulfonic acid (MES) and 4 mM CaCl_2_. All assay components were prewarmed for 20 min at 37°C. The reaction mixture, in a total volume of 100 μl, was incubated at 37°C, and the fluorescence of the enzyme-cleaved 4-methylumbelliferone (4-MU) product was measured every 2 min for 60 min using an SH-9000 lab microplate reader (Corona Electric) with excitation and emission wavelengths of 350 nm and 460 nm, respectively. A standard curve was generated using 4-MU (Sigma-Aldrich) diluted in enzyme buffer to a final concentration of 0.1 to 50 μM and used to convert the fluorescence values to 4-MU concentrations (in micromolar). The NA enzymatic kinetic parameters, the Michaelis-Menten constant (*K_m_*) and the maximum velocity of substrate cleavage (*V*_max_), were calculated with GraphPad Prism (version 6) software (GraphPad) by fitting the data to the Michaelis-Menten equation using nonlinear regression.

### Virus elution assays.

The ability of NA to elute viruses bound on erythrocytes was assayed as described previously (59, 60), with some modifications. Samples (50 μl) of 2-fold dilutions of 128 HAU of each purified virus were incubated with 50 μl of 0.5% chicken or turkey erythrocytes in 96-well round-bottomed microtiter plates at 4°C for 1 h, which allowed the viral HA to bind erythrocyte sialylglycan. The microtiter plates were then transferred to 33°C, which allowed the viral NA to cleave Sia, and the HAU titer was assayed for 60 h. Turkey erythrocytes, which have more α2,6 Sia than α2,3 Sia, were also treated with α2,3 neuraminidase S (New England Biolabs), according to the manufacturer’s instructions, to produce erythrocytes with only α2,6 Sia on their cell surface. PBS containing 6.8 mM CaCl_2_ was used as the diluent. Elution data were plotted against the incubation time at 33°C and analyzed using Origin (version 9.1) data analysis and graphing software (Origin Lab). The data were fitted with the sigmoidal Boltzmann equation, and the elution time at which the HAU titer was reduced from 128 to 64 HAU was calculated. The elution times were expressed relative to the NA-wt elution time, which was set at 100%.

### Viral infection of cultured cells and eggs.

DF-1 and Calu-3 cells (90% confluent in collagen-coated 24-well plates) were infected in triplicate with the indicated viruses at an MOI of 0.005 or 0.03, respectively. After 1 h at 37°C, the DF-1 and Calu-3 cells were washed with PBS and maintained in Dulbecco modified Eagle medium–Ham’s F-12 medium (DMEM-F12) containing 0.2% bovine serum albumin (BSA) and 2 μg acetylated trypsin/ml (Sigma-Aldrich) at 33°C or 37°C, respectively. The supernatants were collected at the times postinfection indicated throughout the text above. Nine-day-old embryonated chicken eggs (5 eggs per group) were inoculated with 1 × 10^5^ FFU of the viruses described above, and allantoic fluids were collected at the times postinoculation indicated throughout the text above. Virus titers were determined by FFU assays on MDCK cells as described above.

### Viral infection of mice.

Four- to 5-week-old female BALB/c mice (Japan SLC) under mixed anesthesia (medetomidine-butorphanol-midazolam) were inoculated intranasally with 50-μl samples of 10-fold serial dilutions (10^3^ to 10^7^ FFU) of viruses in PBS. The body weight and survival of each mouse were monitored daily for 14 days. Mice that lost more than 30% of their original weight within a few days were humanely euthanized. The lungs of mice infected with 10^5^ FFU of virus were collected at 4 dpi, and virus FFU titers were assayed.

### Receptor specificity assays.

HA receptor binding specificity was analyzed by solid-phase binding assays with the Sia analogues Neu5Acα2,3Galβ1-4GlcNAc (α2,3 SLN) and Neu5Acα2,6Galβ1-4GlcNAc (α2,6 SLN), as previously described (23, 51, 52), and α2,3 SLN sulfated on its antepenultimate sugar [Neu5Acα2,3Galβ1-4(6-HSO_3_)GlcNAc (Su-α2,3 SLN); GlycoNZ]. Anti-influenza virus NP antibody was used as the primary antibody, and peroxidase-conjugated goat anti-immunoglobulin (Histofine simple stain MAX-PO; Nichirei) was used as the secondary antibody. The absorbance was measured at a wavelength of 630 nm.

### Quantitative analysis of foci.

Semiconfluent cell monolayers in 96-well plates were inoculated with the viruses indicated above at an MOI of 0.75, which was determined by FFU assays on MDCK cells. After 1 h of adsorption followed by washing with PBS, the cells were overlaid with 1% methylcellulose in modified Eagle’s medium without trypsin, to prevent the release and spread of progeny virus, thereby enabling the observation of foci that resulted from the initial virus infection. After 8 h of incubation at 37°C, the cells were fixed and analyzed by immunofluorescence assays to quantify foci that resulted from the initial virus infection and Hoechst-stained nuclei. Images were captured with an IN Cell analyzer 2000 automated microscope (GE Healthcare). Fields containing both virus antigen-positive cells and cell nuclei were counted by IN Cell Developer Toolbox software (GE Healthcare), as described previously (37). Infection rates were calculated by dividing the number of infected cells by the total number of nuclei in the same field.

### Electron microscopy.

MDCK cells were infected with the viruses indicated above at an MOI of 3. After 18 h of incubation in DMEM-F12 containing 0.2% BSA and 2 μg acetylated trypsin/ml (Sigma-Aldrich), the cells were chemically fixed as described previously (18). After fixation, the samples were dehydrated, embedded in a resin (Quetol-812; Nisshin EM), and thin sectioned, and ultrathin 70-nm sections were stained with 2% uranyl acetate, followed by secondary staining with a lead stain solution (Sigma-Aldrich). After sample preparation, progeny virus particles were observed at 100 kV with a transmission electron microscope (model JEM-1400Plus; JEOL). Digital images were captured by a charge-coupled-device camera (model EM-14830RUBY2; JEOL) and visualized using Adobe Photoshop software (Adobe Systems).

### Statistical analysis.

Statistical analysis was carried out using GraphPad Prism (version 6) software (GraphPad Software). Statistically significant differences between virus pairs were determined by analysis of variance with Tukey’s multiple-comparison test.

### Data availability.

The sequences of the HA and NA gene segments in the CL42, CL79, CL80, and CL83 strains in this study have been deposited in the GenBank database under accession numbers LC379966, LC379968, and LC534257 to LC534262.
